# First Report of On-Site Detection of Cucurbit Leaf Crumple Virus by an Optimized RPA-Lateral Flow Assay with an Alternative Endonuclease

**DOI:** 10.3390/ijms262110611

**Published:** 2025-10-31

**Authors:** A. Abdul Kader Jailani, Mathews L. Paret

**Affiliations:** 1 North Florida Research and Education Center, University of Florida, Quincy, FL 32351, USA; 2 Plant Pathology Department, University of Florida, Gainesville, FL 32611, USA

**Keywords:** RPA-LFT, endonuclease, CuLCrV, begomovirus, whitefly, isothermal, filed-deployable assay

## Abstract

Rapid, simple, and robust diagnostics are essential for effectively controlling the spread of plant viruses and mitigating their impact. Although recombinase polymerase amplification-lateral flow test (RPA-LFT) diagnostics currently offer high sensitivity and specificity, they rely on the Nfo endonuclease enzyme and require an expensive heat block. In this study, we present the development of a molecular diagnostic test for cucurbit leaf crumple virus (CuLCrV) using an RPA-LFT assay that employs an alternative endonuclease enzyme instead of Nfo. This alternative endonuclease demonstrates comparable functionality to Nfo and achieves a detection limit of 10 viral copies in plant samples and whiteflies. The assay can be performed using a battery-powered mini heat block, ensuring scalability and cost-effectiveness. Notably, the unavailability of commercially accessible Nfo endonuclease enzymes underscores the necessity for an alternative enzyme for RPA-LFT assay development. The RPA-LFT assay eliminates the need for nucleic acid purification and provides results within approximately 30 min from sample collection. The integration of this new endonuclease into the RPA-LFT assay represents an advancement towards on-site detection of plant viruses, enabling early-stage management of viral infections.

## 1. Introduction

Cucurbit leaf crumple virus (CuLCrV) is a plant virus that belongs to the family Geminiviridae [1]. It is transmitted by whiteflies and infects a wide range of cucurbit crops, including cucumber, watermelon, and squash [2,3]. CuLCrV infection causes severe symptoms, such as leaf crumpling, yellowing, and stunting, which can result in significant yield losses [4,5,6]. CuLCrV is considered a major threat to the cucurbit industry in the United States and has been reported in many states, including Florida, California, Georgia, Texas, South Carolina, and [4,7,8,9]. Early detection and management of CuLCrV are critical for preventing the spread of the disease and minimizing its economic impact on agriculture. Traditional detection methods are available, such as PCR [10], restriction fragment length polymorphism (RFLP) followed by PCR [11], nucleic acid hybridization [12], and rolling circle amplification (RCA) [13] are effective but require expensive equipment and skilled technicians, which limit their use in field settings. ELISA is a rapid and sensitive technique that can detect viral proteins or antibodies in infected plant tissues [14]. However, this method requires specialized equipment and reagents, and it may not be suitable for field-based diagnostics.

PCR is a molecular technique that amplifies the viral DNA or RNA sequences using specific primers, followed by detection through gel electrophoresis or hybridization. Although PCR is highly sensitive and specific, it requires expensive equipment and trained personnel, making it challenging for use in resource-limited settings. The traditional detection methods have their advantages and limitations, and the choice of the method depends on the specific needs and resources available for the detection of CuLCrV. There are several isothermal methods available for the detection of CuLCrV, including Loop-mediated isothermal amplification (LAMP), LAMP is another isothermal amplification method that uses a set of primers that recognize six distinct regions of the target DNA sequence, leading to the amplification of the target sequence. LAMP operates at a constant temperature, typically 60–65 °C, and can be completed within an hour [15].

Recently, the development of rapid and accurate diagnostic assays based on isothermal amplification technologies, such as RPA, has provided an alternative approach for virus detection. RPA is a robust and sensitive nucleic acid amplification method that operates at a constant temperature and can be completed within 30 min. Additionally, RPA assays can be performed using simple equipment and minimal technical expertise, making it a promising tool for field-based diagnostics [16]. More recently, a singleplex exo RPA assay has been developed for the detection of CuLCrV under field-based conditions, utilizing a battery-operated portable spectrometer [17]. Additionally, a lab-based multiplex conventional RPA assay has been developed for three cucurbit viruses, including CuLCrV [18]. While the exo RPA assay offers a field-based diagnostic method, it necessitates a field-operable spectrophotometer, which can be more expensive. Conversely, the lab-based RPA assay is not applicable under field-based conditions.

Furthermore, previously reported assays have all relied on instruments for virus detection, and none have been utilized for the detection of CuLCrV using the RPA-LFT (Recombinase Polymerase Amplification-Lateral Flow Test) assay. The RPA for nucleic acid amplification, followed by detection on a lateral flow test strip, enables rapid and sensitive detection of target pathogens in a user-friendly format. Rapid and reliable detection of plant viruses is essential for effective disease management, particularly in resource-limited or field settings. Traditional diagnostic methods, such as PCR and ELISA, are sensitive and specific but require sophisticated equipment and trained personnel, limiting their use outside laboratory environments. The RPA assay, when coupled with a LFT, provides a rapid, sensitive, and instrument-free method for detecting plant viruses such as CuLCrV. In this study, we introduce the use of Thermus thermophilus (Tth) endonuclease, a thermostable enzyme that enhances probe cleavage efficiency during RPA amplification, thereby improving the sensitivity and visual clarity of the RPA-LFT assay for field-based virus diagnostics. Unlike commonly used nucleases, Tth endonuclease functions efficiently at elevated temperatures and demonstrates robust activity under field-like conditions. Its ability to specifically cleave at designated sites enhances signal generation and contributes to a clearer, stronger visual output on the lateral flow strip.

**Figure 1:**
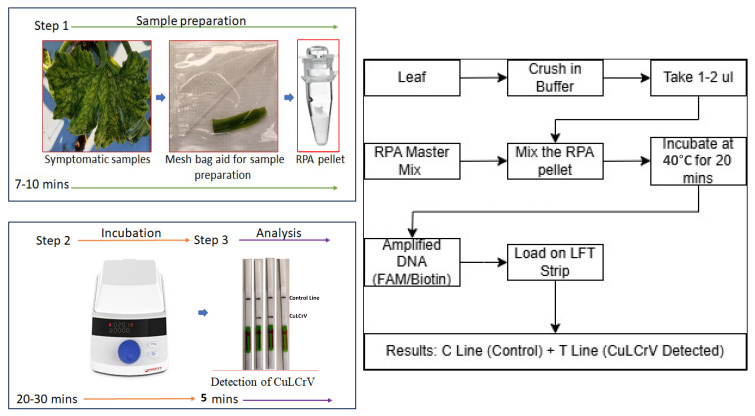
Workflow of the RPA-LFT assay for detection of CuLCrV. Symptomatic leaf tissues from cucurbit plants were processed for nucleic acid extraction. RPA was carried out at 40 °C using CuLCrV-specific primers and probes. Amplified products were applied to Milenia HybriDetect lateral flow strips for visualization. A positive result was indicated by the presence of both control (°C) and test (T) Test lines, while a negative result showed only the control line. The entire assay was completed within 30–40 min, providing a rapid, field-deployable diagnostic method for CuLCrV.

In this study, we developed a new RPA-based lateral flow test (LFT) assay for the detection of CuLCrV using the Tth endonuclease. This LFT assay is a user-friendly, rapid, and cost-effective diagnostic tool that combines the amplification and detection of the target nucleic acid in a single reaction. The LFT assay employs gold nanoparticle-conjugated probes that bind to the amplified product and produce a visible signal on the test strip. This signal can be easily read by the naked eye, eliminating the need for specialized equipment or trained personnel. The objective of this study was to evaluate the sensitivity and specificity of the RPA-based LFT assay for the detection of CuLCrV, and to compare its performance with that of traditional detection methods. The results of this study further demonstrate the potential of RPA-based LFT assays as a rapid and reliable diagnostic tool for the detection of CuLCrV in field setting, which can aid in the effective management and control of this destructive plant virus. The figure and flowchart’s schematic representations are explained in the field-based RPA-LFT assay development of CuLCrV detection (Figure 1).

## 2. Results

### 2.1. CuLCrV Detection in Plant and Whitefly Samples by PCR and qPCR

The coat protein amplification was observed in 25 samples (92.6%), with bands corresponding to the expected ~370 bp product. No amplification was detected in healthy samples and negative controls. DNA extracts exhibited good purity (A260/280 ratios between 1.8 and 2.0), ensuring suitability for downstream applications [19]. PCR detection of CuLCrV was also conducted in 15 whitefly (*B. tabaci*) samples. All samples (100%) produced positive amplification, indicating the consistent presence of the virus in the vector population and supporting the method’s diagnostic robustness for insect-derived DNA [20]. Quantitative real-time PCR (qPCR) identified CuLCrV in 25 of the 27 cucurbit samples (92.6%). Positive samples showed characteristic sigmoidal amplification curves with cycle threshold (Ct) values confirming the presence of the target. Additionally, no signal was detected in negative controls or healthy samples [20]. All 15 whitefly samples were positive for CuLCrV by qPCR, yielding consistent Ct values across replicates. These results demonstrate the high sensitivity and specificity of the qPCR assay for detecting CuLCrV in insect vectors [20].

### 2.2. CuLCrV Detection in Plant and Whitefly Samples by RPA

**Figure 2:**
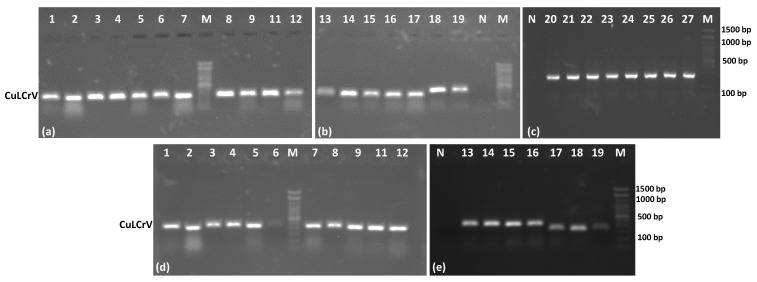
Detection of CuLCrV in plant and whitefly samples using the conventional RPA assay. (**a**–**c**) Amplified products from symptomatic cucurbit plant samples (watermelon L-1, squash L, and pumpkin) collected from the NFREC field in Quincy, FL. (**d**,**e**) Amplified products from whitefly samples collected from infected plants. Each lane represents an individual sample; M: molecular marker; N: negative control.

The RPA assay was successfully used to detect CuLCrV in 27 of the 27 cucurbit samples (100%) under isothermal conditions (40 °C for 40 min). Amplification products produced clear, expected-size bands on agarose gels (Figure 2a–c). No amplification was observed in healthy plant samples or no-template controls, confirming the assay’s specificity (Figure 2b,c). All whitefly samples tested positive using the RPA assay, confirming 100% detection (Figure 2d,e). The high consistency of results across replicates supports the use of RPA for rapid virus detection in vector insects.

### 2.3. Optimization of Endonuclease Enzyme Performance for RPA-LFT

**Figure 3:**
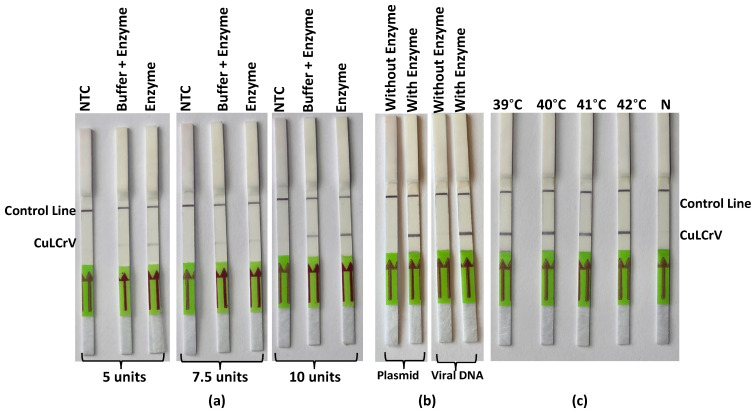
Optimization of Tth endonuclease enzyme and temperature validation in a RPA-LFT assay. (**a**) Optimization of exonuclease enzyme combinations for improved signal clarity in the RPA-LFT assay. (**b**) Evaluation of enzyme performance with and without RPA-LFT assay components using CuLCrV DNA and artificial plasmid control as templates. (**c**) Temperature optimization for RPA-LFT assay performance using exonuclease enzyme, showing the influence of incubation conditions on detection sensitivity. NTC: negative control. Arrows indicate the direction of sample flow toward the test (T) and control (C) lines.

To improve the visual clarity and reliability of the RPA-LFT assay, different concentrations of endonuclease enzyme units were evaluated (Figure 3). Among the tested conditions, 10 units of Tth endonuclease produced the most distinct and reproducible test and control lines, indicating enhanced cleavage activity and improved signal development efficiency (Figure 3a). In contrast, reactions with 5 and 7.5 units yielded weaker test lines, suggesting suboptimal enzyme activity under those conditions (Figure 3a). This comparative analysis identified 10 units as the optimal concentration for signal generation in the RPA-LFT assay. Additionally, no significant differences were observed between reactions containing enzyme alone and those with enzyme plus buffer, indicating that buffer supplementation did not influence assay performance in RPA-LFT master mix.

### 2.4. Validation of Enzyme Activity with Template Types

Enzyme activity was further validated by assessing the presence and absence of Tth endonuclease in the RPA-LFT assay using complete reaction components, including both plasmid and DNA controls (Figure 3). Strong and consistent test lines were observed in reactions containing Tth endonuclease along with all assay components (Figure 3b), whereas reactions lacking the enzyme showed no visible test lines (Figure 3b). These results confirm the assay’s specificity and validate the functional activity of Tth endonuclease in facilitating signal generation.

### 2.5. Temperature Optimization for Endonuclease-Based RPA-LFT

To determine the optimal incubation temperature for the endonuclease-based RPA-LFT assay, reactions were conducted at various temperatures (Figure 3c). The assay produced strong signal intensity and clear band development across all tested temperatures. However, 40 °C yielded the most consistent and robust results, confirming it as the optimal temperature for sensitive and reliable detection of CuLCrV using this assay format.

### 2.6. Development and Validation of the RPA-LFT Assay for CuLCrV Detection in Plant and Whitefly Samples

**Figure 4:**
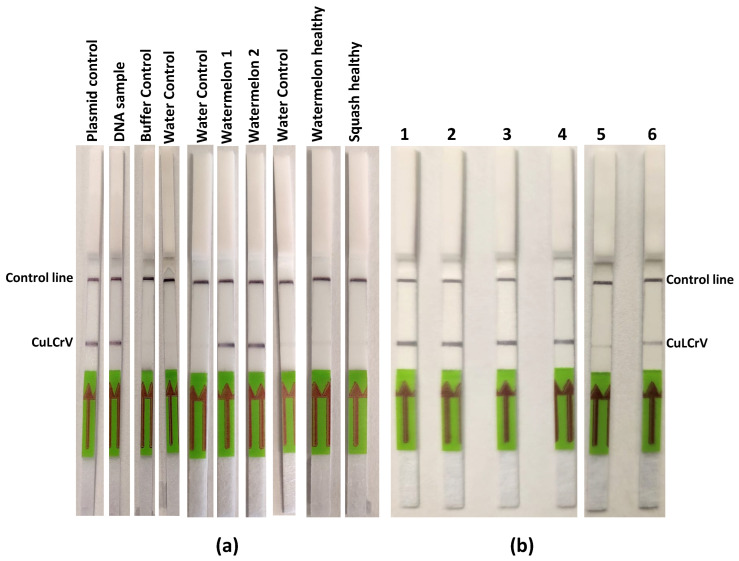
Development and validation of the RPA-LFT assay for the detection of CuLCrV using plant and whitefly samples. (**a**) Lateral flow test strips showing amplification results from CuLCrV-infected plant tissue samples. (**b**) Lateral flow test strips using DNA templates from whiteflies samples (lanes 1–4) and direct crude whitefly extracts (lane 5: single whitefly, lane 6: ten whiteflies) as input for the RPA assay. Arrows indicate the direction of sample flow toward the test (T) and control (C) lines.

To evaluate the diagnostic performance of the developed RPA-LFT assay using a new Tth endonuclease, both CuLCrV-infected plant tissues and whitefly samples were tested under field-relevant conditions (Figure 4). As shown in Figure 4a, the RPA-LFT assay successfully detected CuLCrV in all symptomatic plant tissue samples, as evidenced by the presence of distinct test and control lines. In contrast, no test lines were observed in the healthy and no-template control samples, although both displayed clear control lines, confirming the validity of the negative results. The assay was further validated using DNA extracted from individual and pooled whitefly samples (lanes 1–4, Figure 4b), as well as crude extracts from a single whitefly (lane 5) and a pool of ten whiteflies (lane 6). Strong test lines were consistently observed across all whitefly-derived samples, including the minimally processed crude extracts, demonstrating the assay’s robustness and suitability for rapid vector surveillance in field settings.

### 2.7. Sensitivity and Specificity of RPA-LFT Assays

**Figure 5:**
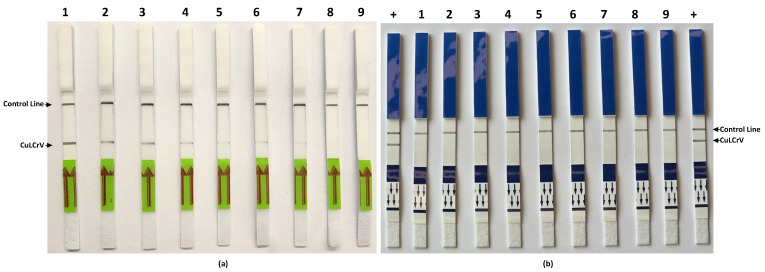
Examination of RPA-LFT assay primers and probe for sensitivity and specificity. (**a**) Sensitivity assay using serial tenfold dilutions of artificial plasmid control (APC), ranging from 10^6^ copies to 1 copy per reaction, showing decreasing signal intensity corresponding to template dilution. (**b**) Specificity assay using nucleic acids from various cucurbit-infecting viruses including 1-CCYV, 2-SqVYV, CYSDV, PRSV-W, WMV, ToLCNDV, ZYMV, and WaLCV1 & WaLCV2, demonstrating no cross-reactivity with non-target viruses. Arrows indicate the direction of sample flow toward the test (T) and control (C) lines.

The sensitivity and specificity of the RPA-LFT assay targeting CuLCrV were evaluated using artificial plasmid controls (APCs) and nucleic acid samples from other cucurbit-infecting viruses. As shown in Figure 5a, the assay successfully detected CuLCrV down to a 10 copies level. A clear test line was visible with all tenfold serial dilutions of APC DNA, ranging from 10^6^ to 10^1^ per reaction, although band intensity progressively decreased with lower template concentrations, indicating high sensitivity and limit of detection at 10 copies. To assess specificity, the assay was tested against nucleic acid samples from eight non-target cucurbit-infecting viruses: CCYV, SqVYV, CYSDV, PRSV-W, WMV, ToLCNDV, ZYMV, and WaLCV1 & 2. As shown in Figure 5b, none of these samples produced visible test lines, confirming the assay’s specificity and absence of cross-reactivity with closely related viruses. These results demonstrate that the developed RPA-LFT assay is both highly sensitive and specific for CuLCrV detection.

### 2.8. CuLCrV Detection in Field Samples by RPA-LFT

**Figure 6:**
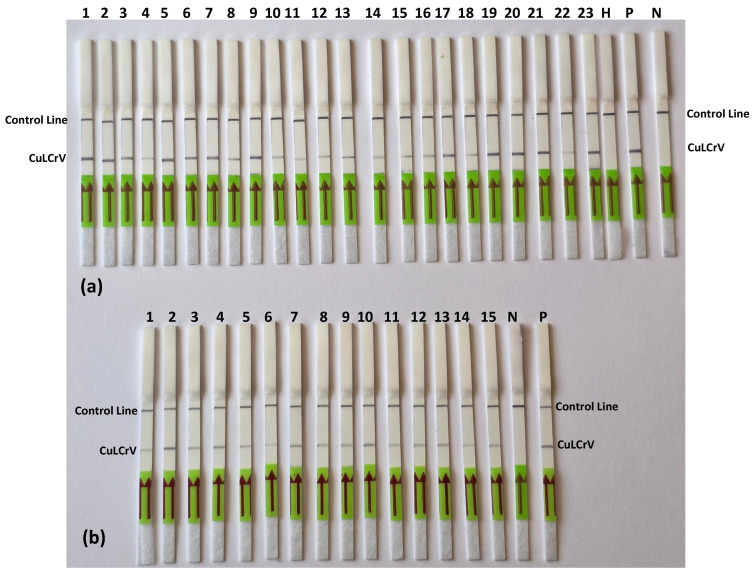
Application of the RPA-LFT assay for detecting CuLCrV in infected plant and whitefly samples. (**a**) Detection of CuLCrV from symptomatic cucurbit plant tissues, including squash (lanes 1–14), watermelon (lanes 15–19, 22–23) and pumpkin (lanes 20–21), using the RPA-LFT assay. (**b**) Detection of CuLCrV from individual and mixed whitefly samples using the same RPA-LFT assay conditions. lanes 1–15: whitefly samples carrying CuLCrV; N: negative control; P: positive control and H: healthy control. Arrows indicate the direction of sample flow toward the test (T) and control (C) lines.

To evaluate the field applicability of the RPA-LFT assay, we tested symptomatic cucurbit plant tissues and whitefly samples collected from CuLCrV-infected sites. As shown in Figure 6a, 27 plant samples—including 14 squash, 5 watermelon, and 3 pumpkin samples—were subjected to RPA amplification followed by lateral flow detection. All 27 samples (100%) produced strong test and control lines, confirming the presence of CuLCrV. In contrast, the negative (N) and healthy (H) controls generated only the control line, demonstrating the specificity of the assay (Table 1). Enzyme optimization showed that the use of Tth polymerase provided more intense and consistent signal bands compared to the standard Nfo enzyme, thereby improving the visual clarity and robustness of test results. In Figure 6b, the RPA-LFT assay was applied to whitefly vector samples. All 15 mixed whitefly samples (lanes 1–15) tested positive for CuLCrV, showing distinct test and control bands within 10 min of incubation (Table 2). No amplification was observed in the negative and healthy controls. These results were fully concordant with PCR, qPCR, and conventional RPA assays performed in parallel, confirming both the diagnostic reliability and field-deployability of the developed RPA-LFT assay for CuLCrV detection in both plant hosts and insect vectors.

## 3. Discussion

Cucurbit leaf crumple virus (CuLCrV) poses a significant threat to cucurbit production across the United States due to its rapid spread, broad host range, and association with severe yield losses [21,22]. Current diagnostic techniques such as PCR, qPCR, ELISA, and nucleic acid hybridization provide reliable virus detection but are often constrained by their dependence on expensive equipment, skilled personnel, and laboratory infrastructure [10,11,12,13,14,15,16,17,18,19]. This limits their deployment in field conditions where timely detection is essential for managing disease outbreaks. Although LAMP assays have improved the feasibility of field diagnostics [15], their complexity in primer design and operation temperature still restricts widespread application.

To address these limitations, we developed and validated a RPA-LFT assay that offers a rapid, sensitive, and equipment-independent method for detecting CuLCrV in both plant and vector samples. RPA is an isothermal amplification method that operates at low temperatures (~40 °C) and can be completed in less than 30 min, making it well suited for field-based diagnostics [23,24]. The lateral flow detection system allows for immediate visual interpretation of results without the need for instrumentation, addressing the current gap in cost-effective, field-deployable assays for CuLCrV [16].

Traditional RPA-LFT assays often utilize endonuclease IV (Nfo) for probe cleavage. However, this study explored the use of Tth endonuclease, a thermostable enzyme known for its robust activity at elevated temperatures [25]. By optimizing the concentration of Tth endonuclease to 10 units per reaction, the assay achieved clearer and more reproducible test and control lines on the lateral flow strips. This enhancement is attributed to Tth endonuclease’s efficient cleavage of the probe, facilitating better signal development. Unlike previous RPA-LFT assays reported for plant viruses, this study is the first to incorporate the thermostable Tth endonuclease as an alternative to Nfo for probe cleavage, enabling improved signal consistency and enhanced performance under field conditions. The thermostability of Tth endonuclease allows the RPA-LFT assay to operate effectively at 40 °C, a temperature conducive to field conditions. This attribute is particularly beneficial for on-site diagnostics, where precise temperature control may be challenging. The successful application of Tth endonuclease in this context underscores its potential utility in other isothermal amplification assays requiring robust enzyme activity under variable environmental conditions.

A key distinction between the present assay and conventional RPA systems lies in the use of Tth endonuclease instead of the commonly employed Endonuclease IV (Nfo). The Tth enzyme exhibits higher thermostability and remains catalytically active across a wider temperature range (35–65 °C), which is advantageous for field conditions where precise temperature control is difficult [24]. In contrast, Nfo is less thermostable and performs optimally below 42 °C, limiting its robustness in warmer environments [16]. Although both enzymes efficiently cleave double-stranded probe intermediates during RPA, our findings demonstrate that Tth-based RPA-LFT maintains comparable sensitivity and reliability to Nfo-based assays while offering greater operational flexibility and cost efficiency for on-site plant virus detection.

Our assay demonstrated high analytical sensitivity, detecting CuLCrV down to 10 copies of plasmid DNA, and exceptional specificity, showing no cross-reactivity with other cucurbit-infecting viruses including CCYV, CYSDV, SqVYV, PRSV-W, WMV, ZYMV, ToLCNDV, WaLCV1, and 2. These findings confirm the robustness of the primer-probe set designed specifically to target a conserved region in the CuLCrV genome and validated through in silico and empirical approaches. We further optimized the assay by testing new endonuclease enzyme and reaction conditions. The use of Tth endonuclease provided stronger, more consistent signal development than Nfo, suggesting that enzyme selection can significantly enhance assay performance. Additionally, 40 °C was found to be the optimal temperature for consistent amplification and detection, confirming earlier studies on RPA assay optimization [26].

In field sample validation, the RPA-LFT assay successfully detected CuLCrV in all 27 symptomatic cucurbit samples (squash, watermelon, and pumpkin), as well as in 15 vector (whitefly) samples. These results were entirely consistent with conventional PCR, qPCR, and standard RPA assays, confirming the diagnostic accuracy of the developed LFT method. Notably, the assay also worked effectively using crude whitefly extracts, eliminating the need for nucleic acid extraction and further streamlining field testing. This adaptability offers a significant advantage for pest surveillance and rapid intervention in infected fields [15].

Importantly, this study is the first report of a Tth-based RPA-LFT assay for CuLCrV detection, and one of the few to demonstrate successful integration of enzyme optimization and field sample testing across plant and insect hosts. By providing an instrument-free, sensitive, and user-friendly diagnostic tool, the RPA-LFT assay represents a practical solution for CuLCrV monitoring in low-resource and remote agricultural settings. The potential of this platform extends beyond CuLCrV. Given the modularity of RPA and the flexibility of lateral flow detection, similar diagnostic formats could be developed for other whitefly-transmitted viruses and emerging plant pathogens. Future directions may include the integration of this assay with smartphone-based readers or lyophilized reagent formats to further enhance portability, shelf life, and accessibility. The recent development of gold nanoparticle-based lateral flow tests (AuNPs-LFTs), an avenue for rapid, high-throughput field diagnostics [27].

## 4. Materials and Methods

### 4.1. Sample Collection and Nucleic Acid Extraction

Samples of infected plants displaying symptoms of CuLCrV were collected from watermelon, pumpkins, and squash plants at the NFREC research fields in Quincy, FL, USA. Representative samples with visible signs such as crumpled leaves, yellowing, and stunted growth were carefully chosen. To prevent any contamination between samples, aseptic techniques and disposable gloves were employed. Each sample was assigned a unique identifier to ensure traceability. To minimize the degradation of viral nucleic acids, the collected plant samples were promptly transported to the laboratory and stored in a cool, dark location. In cases where immediate processing was not possible, the samples were stored at −80 °C until further analysis. The Quick-DNA Plant Mini Kit from Qiagen (Redwood City, CA, USA) was utilized to extract total nucleic acids from the infected plant samples, following the manufacturer’s instructions. The extracted nucleic acids were evaluated for quality, integrity, concentration, and purity using a Nano Spectrometer from Thermo Scientific (Waltham, MA, USA). The presence of CuLCrV was confirmed by conducting a conventional PCR with specific primers targeting the CP region, as described by Jailani et al. (2021) [19]. Gel electrophoresis was employed to visualize the amplified CP region and confirm the presence of the target product. The extracted nucleic acids were stored at −80 °C for further validation and the RPA-LFT assay.

### 4.2. Crude Extract Preparation from Plant Tissue Samples

Fresh leaf tissue (approximately 100 mg, equivalent to a small leaf disc or a 1 cm^2^ section) was placed in a disposable extraction bag containing 1000 µL of GBE 1 extraction buffer. The tissue was manually ground using the integrated roller of the extraction bag until a uniform homogenate was obtained. The crude extract was then allowed to settle for 1–2 min at room temperature. A 1–2 µL aliquot of the clear supernatant was immediately used as the template for the RPA reaction without further purification. This simple, rapid extraction approach minimized processing time and was suitable for field-based molecular detection assays.

### 4.3. Nucleic Acid Extraction from Whiteflies

For the extraction of whitefly nucleic acids, ethanol-preserved adult whiteflies (*Bemisia tabaci*) were first washed twice with sterile double-distilled water to remove external contaminants. Excess water was carefully removed, and the insects were air-dried on sterile filter paper. The dried whiteflies were then homogenized in sterile 2 mL microcentrifuge tubes using an autoclaved pestle. A minor modification was introduced to the standard extraction protocol—30 µL of extraction buffer was used in the initial step instead of the recommended 450 µL to accommodate the small tissue volume. Both single and pooled whitefly samples were homogenized in 30 µL of the modified extraction buffer supplied with the RNeasy Plant Mini Kit (Qiagen, Hilden, Germany). Total nucleic acids were then extracted following the same procedure used for plant tissue samples, as described by Jailani et al. (2024) [20].

### 4.4. RPA Primer and RPA-LFT Probe Design for CuLCrV Detection

In order to design primers and probe for CuLCrV, a specific region (coat protein) within the CuLCrV genome was identified. This region needed to exhibit high conservation and be appropriate for primer design. It was essential for the selected target region to be unique to CuLCrV and possess minimal sequence similarity to other viruses that infect cucurbits or cucurbit genomes. Bioedit software 7.7 was utilized to design primers that specifically bind to the chosen target region, ensuring a high level of specificity for CuLCrV detection. The primers and probe were designed following the TwistDx manufacturer’s protocol for the RPA assay, adhering to the recommended length of 30–35 base pairs for primers and 46–54 base pairs for probe and achieving a melting temperature (Tm) suitable for RPA conditions. To validate the RPA primers and RPA-LFT probe, in silico analysis was conducted against known CuLCrV sequences and other closely related viruses to assess their specificity. Additionally, an analysis of the primer and probe sequences were performed against the NCBI GenBank database to avoid any potential cross-reactivity. The RPA primers were synthesized by Integrated DNA Technology (Skokie, IL, USA) as per manufacture protocol. The RPA-LFt probe was synthesized by LGC Biosearch Technologies (Petaluma, CA, USA) (Table 3).

### 4.5. Conventional RPA Reaction

The RPA reaction was conducted to prepare a 50-µL reaction volume, following the guidelines provided in the RPA-basic kit (TwistDx, Cambridge, UK). The reaction mixture consisted of 29.5 µL of rehydration buffer, 2.1 µL of forward primer, 2.1 µL of reverse primer (both at a concentration of 10 µM), a single lyophilized enzyme pellet, 1 µL of DNA template (plant 10–50 ng and whitefly 2–10 ng), and 12.8 µL of nuclease-free water. The reaction was initiated by adding 2.5 µL of 280 mM magnesium acetate. After an incubation period of 40 min at 40 °C, the amplicons were purified using the Qiagen PCR purification Kit (Qiagen, Hilden, Germany), following the manufacturer’s protocol, and subsequently subjected to electrophoresis on 2.0% agarose gels.

### 4.6. Optimization of Endonuclease Enzyme Performance for RPA-LFT

To determine the optimal concentration of endonuclease enzyme for the RPA-LFT assay, different units (5, 7.5, and 10 units) of *Thermus thermophilus* (Tth) (Cat. No. M0294S, NEB, Ipswich, MA, USA) endonuclease were tested in the reaction mixture. Each reaction was prepared with TwistAmp^®^ Basic kit (TwistDx, Maidenhead, UK) components, probe (10 μM) and amplification results were evaluated based on test line intensity and consistency on lateral flow strips (HybriDetect-Universal Lateral Flow Assay Kit, Milenia Biotec GmbH, Giessen, Germany). In parallel, the effect of buffer addition with the enzyme was assessed by preparing reactions with enzyme alone and enzyme plus buffer to determine any influence on signal development.

### 4.7. Validation of Endonuclease Activity with Different Template Types

To confirm the functionality of Tth endonuclease in the RPA-LFT assay, reactions were prepared using both CuLCrV-infected plant DNA and plasmid DNA as templates. Parallel reactions were performed with and without the addition of endonuclease to evaluate its necessity in signal generation. Lateral flow test strips were used to detect amplification products, and visual signal presence or absence was recorded to assess assay specificity and enzyme activity.

### 4.8. Temperature Optimization for Endonuclease-Based RPA-LFT Assay

To identify the optimal incubation temperature for the endonuclease-based RPA-LFT assay, reactions were incubated at different temperatures ranging from 39 °C to 42 °C with a battery-powered mini heat block. All reactions included the full set of RPA-LFT reagents and 10 units of *Tth* endonuclease. After amplification, lateral flow strips were used to visualize the results, and the clarity and intensity of test lines were compared across the tested temperatures to establish optimal conditions for sensitive detection of CuLCrV.

### 4.9. RPA-LFT Assay Development

Designing primers and probes for the RPA-LFT assay involves identifying conserved regions in the target pathogen, such as CuLCrV, and designing specific primers and probes according to the Twist DX, UK protocol. The designed primers and probes are then validated for strength and specificity using bioinformatics software such as BioEdit 7.7. The RPA reaction for the LFT assay was conducted under the same reaction conditions as mentioned previously. Additionally, for the RPA reaction in the LFT assay, *Tth* (1 μL/reaction) (NEB, Ipswich, MA, USA), were added as alternatives to the *Nfo* enzyme performances. The enzyme units were optimized at different levels, and we utilized the final standardized units in the above RPA-LFT assay. In the RPA assay, the probe and primer concentrations were adjusted slightly according to the manufacturer’s protocol used for the RPA-LFT assay with the TwistDX *Nfo* kit.

### 4.10. Lateral Flow Test (LFT) Visualization

Following the completion of the CuLCrV RPA assay, the amplified products were analyzed using Milenia HybriDetect lateral flow test (LFT) strips for the detection of biotin- and FITC-labeled analytes. According to the manufacturer’s protocol, a 5 μL volume of the CuLCrV RPA amplification product was directly pipetted onto the sample application area of the Milenia^®^ HybriDetect LFT strip. Subsequently, the test strips were immersed in a tube containing 100 μL of HybriDetect Assay Buffer and incubated for 5–15 min to observe the results.

### 4.11. Sensitivity and Specificity Evaluation

To evaluate the sensitivity of the RT-RPA assay and compare it with the conventional RPA assay, we utilized tenfold serial dilutions (10^0^ to 10^6^) of an artificial plasmid control (APC) as the template for this sensitivity assessment. Subsequently, these APCs were subjected to analysis using conventional PCR. For assessing the specificity of the CuLCrV RPA-LFT assay, we investigated potential cross-reactivity with common cucurbit-infecting viruses, including cucurbit yellow stunting disorder virus (CYSDV), cucurbit chlorotic yellows virus (CCYV), squash vein yellowing virus (SqVYV), papaya ringspot virus-watermelon strain (PRSV-W), watermelon mosaic virus-2 (WMV-2), and watermelon crinkle leaf-associated viruses 1 & 2 (WCLaV1). The primers and probe designed specifically for CuLCrV in this study were utilized for this assessment to ensure the assay’s specificity. PCRD FLEX duplex LFT strips (Cytiva, Amersham, UK) were used for specificity assays due to supply delays, while Milenia HybriDetect single-plex strips were used for other tests. The appearance of test and control lines indicated positive and valid reactions, respectively.

## 5. Conclusions

This study reports, for the first time, the successful development and validation of a Tth endonuclease-based RPA-LFT assay for the rapid detection of CuLCrV in both plant tissues and insect vectors. The novel incorporation of Tth endonuclease into the RPA-LFT platform significantly enhanced visual signal clarity and detection consistency compared to traditional enzyme systems such as endonuclease IV (Nfo), offering a substantial improvement in the assay’s diagnostic performance. The RPA-LFT assay exhibited excellent analytical sensitivity, detecting as few as 10 copies of the target DNA, and demonstrated complete specificity, with no cross-reactivity observed with closely related cucurbit-infecting viruses. Importantly, the assay produced reliable results using both purified DNA and crude extracts, underscoring its adaptability for field-based diagnostics without the need for complex nucleic acid extraction protocols or sophisticated equipment.

The portability, rapid turnaround time (results within 10–15 min), and user-friendly nature of this assay make it especially suitable for on-site deployment in cucurbit-growing regions where early detection and rapid response are critical for managing CuLCrV outbreaks. By enabling timely and accurate virus identification at the point of need, this assay has the potential to greatly enhance integrated pest and disease management strategies, support quarantine and surveillance programs, and ultimately mitigate the economic losses associated with CuLCrV infections. Overall, the *Tth* endonuclease–optimized RPA-LFT assay represents a significant advancement in isothermal plant virus diagnostics. Its robust performance and field-applicability make it a promising diagnostic platform not only for CuLCrV, but also for adaptation to other agriculturally important plant pathogens. Future integration with lyophilized reagents and lateral flow devices may further streamline its use in resource-limited and remote agricultural settings.

## Figures and Tables

**Table 1:** Comparison of CuLCrV detection across PCR, qPCR, RPA, and RPA-LFT assays in field samples collected from different hosts in Quincy, FL.

Sample No	Sample Name	Host	Virus Positive for			
			PCR	qPCR	RPA	RPA-LFT
1	101	Sq	+	+	+	+
2	102	Sq	+	+	+	+
3	103	Sq	+	+	+	+
4	104	Sq	+	+	+	+
5	105	Sq	+	+	+	+
6	106	Sq	+	+	+	+
7	107	Sq	+	+	+	+
8	108	Sq	+	+	+	+
9	109	Sq	−	−	+	+
10	110	Sq	+	+	+	+
11	111	Sq	−	−	+	+
12	112	Sq	+	+	+	+
13	113	Sq	+	+	+	+
14	114	Sq	+	+	+	+
15	363	WA	+	+	+	+
16	364	WA	+	+	+	+
17	365	WA	+	+	+	+
18	366	WA	+	+	+	+
19	367	WA	+	+	+	+
20	368	WA	+	+	+	+
21	370	WA	+	+	+	+
22	Kat1	WA	+	+	+	+
23	Kat2	WA	+	+	+	+
24	Kat3	WA	+	+	+	+
25	PM1	PM	+	+	+	+
26	PM2	PM	+	+	+	+
27	PM3	PM	+	+	+	+
28	Negative/NTC	−	−	−	−	−
29	Healthy	Sq/WA	−	−	−	−

Sq: Squash, WA: Watermelon, PM: Pumpkin, PCR: Conventional polymerase chain reaction, qPCR: Quantitative real-time PCR, RPA: Recombinase polymerase amplification, RPA-LFT: RPA combined with lateral flow test, CuLCrV: cucurbit leaf crumple virus, +: positive, −: negative, NTC: no template control.

**Table 2:** Detection of CuLCrV in whiteflies collected from symptomatic plant samples using PCR, qPCR, RPA, and RPA-LFT assays.

Sample No	Sample Name	Virus Positive for			
		PCR	qPCR	RPA	RPA-LFT
1	WF1	+	+	+	+
2	WF2	+	+	+	+
3	WF3	+	+	+	+
4	WF4	+	+	+	+
5	WF5	+	+	+	+
6	WF6	+	+	+	+
7	WF7	+	+	+	+
8	WF8	+	+	+	+
9	WF9	+	+	+	+
10	WF10	+	+	+	+
11	WF11	+	+	+	+
12	WF12	+	+	+	+
13	WF13	+	+	+	+
14	WF14	+	+	+	+
15	WF15	+	+	+	+
NTC/H	WF	−	−	−	−

WF: Whitefly, PCR: Conventional polymerase chain reaction, qPCR: Quantitative real-time PCR, RPA: Recombinase polymerase amplification, RPA-LFT: RPA combined with lateral flow test, CuLCrV: Cucurbit leaf crumple virus, +: positive, −: negative, NTC: no template control, H: healthy.

**Table 3:** Details of the primers and probe used for Conventional PCR, qPCR, Conventional RPA, and RPA-LFT for the detection of CuLCrV.

Name	Assay		Sequences	Dye	Reference
MP5F	PCR	CuLCrV-CP-F	GTTTCCCGCTCTGCTAACTATAC	NA	Jailani et al., 2021 [19]
MP6R		CuLCrV-CP-R	TATACGGTCGACGGTCTCTTAC		
MP11F	qPCR	CuLCrV-qPCR-F	AAGGTCACTGGTGGTCAATATG	FAM	Jailani and Paret 2024 [20]
MP12P		CuLCrV-qPCR-P	AACGAGCAAGCCTTAGTTAGGCGA		
MP13R		CuLCrV-qPCR-R	CTGCTTCCTGGTGGTTGTAG		
MP71	RPA	CuLCrV-RPA-F	CCGTCGACCGTATAGTTCTCCTATGGATTTC	NA	Jailani and Paret 2023 [18]
MP72		CuLCrV-RPA-R	CATGCCATATACAATAACAAAGCGTTCTCAGTATG		
MP71	RPA-LFT	CuLCrV-RPA-F	CCGTCGACCGTATAGTTCTCCTATGGATTTC		NA
MP145		CuLCrV-LFT-Probe	[5′-Label-FAM] ACAGGTTCTATGCAAAGGTCACTGGTGGTC[THF]ATATGC GAGCAACGA[SPC03]	FAM	
MP146		CuLCrV-LFT-R-biotin	[5′-Label-Biotin] CATGCCATATACAATAACAAAGCGTTCTCAGTATG	Biotin	

CP: coat protein; CuLCrV: cucurbit leaf crumple virus; P: probe; LFT: lateral flow test; F: forward primer; R: reverse primer; FAM: 6-carboxyfluorescein; THF: tetrahydrofuran abasic–site mimic.

## Data Availability

The original contributions presented in this study are included in the article. Further inquiries can be directed to the corresponding authors.
